# Satisfaction With Telehealth Services Compared With Nontelehealth Services Among Pediatric Patients and Their Caregivers: Systematic Review of the Literature

**DOI:** 10.2196/41554

**Published:** 2023-04-27

**Authors:** Gergana Damianova Kodjebacheva, Taylor Culinski, Bushra Kawser, Katelynn Coffer

**Affiliations:** 1 Department of Public Health and Health Sciences College of Health Sciences University of Michigan-Flint Flint, MI United States; 2 International Institute University of Michigan Ann Arbor, MI United States; 3 Department of Behavioral Sciences College of Arts and Sciences University of Michigan-Flint Fint, MI United States

**Keywords:** satisfaction, pediatrics, telehealth, telemedicine, virtual care, caregivers, patients, children, COVID-19, coronavirus, SARS-CoV-2, technology use, caregiver, adolescent, youth, satisfaction survey, health outcome, review methodology, systematic review

## Abstract

**Background:**

Telehealth refers to the use of technology to deliver health care remotely. The COVID-19 pandemic has prompted an increase in telehealth services.

**Objective:**

This study aimed to review satisfaction with pediatric care in studies that had at least one group of pediatric patients and their caregivers receiving telehealth services during the COVID-19 pandemic and at least one comparison group of those receiving nontelehealth services.

**Methods:**

We searched for peer-reviewed studies published in the English language that compared the satisfaction with pediatric care between pediatric patients and their caregivers receiving telehealth services during the COVID-19 pandemic and those receiving nontelehealth services. Owing to stay-at-home orders, studies with comparison groups for nontelehealth services that took place either before or during the pandemic were eligible. We searched the PubMed, Embase, CINAHL, and PsycINFO databases on January 5, 2023. We followed the PRISMA (Preferred Reporting Items for Systematic Reviews and Meta-Analyses) guidelines. A total of 2 reviewers independently screened the titles and abstracts before reviewing the full text of the remaining articles. The following information was extracted from each eligible study: country, participant characteristics by comparison group, study design, telehealth approach, measurement tools to assess satisfaction, and findings by comparison group.

**Results:**

All 14 eligible studies assessed satisfaction among caregivers and pediatric patients participating in video or telephone visits during the COVID-19 pandemic compared with those having in-person appointments either before or during the pandemic. In 5 of the 14 studies, a comparison of nontelehealth services took place before the pandemic, and in the remaining 9 investigations, nontelehealth services took place during the pandemic. A total of 13 studies were observational investigations with different designs, and 1 study was a quasi-experimental intervention with 3 comparison groups for video, in-person, and hybrid visits. In 9 of the 14 studies, satisfaction with telehealth services was higher than during in-person visits. Caregivers were satisfied with video visits for the ease of use and reduced need for transportation. Reasons caregivers were not satisfied with remote care included limited personal interaction with the provider, technological challenges, and a lack of physical examination. Those participating in nontelehealth services expressed that in-person interactions promoted treatment adherence. Only 1 study assessed satisfaction where adolescent patients completed their own surveys; a higher percentage of adolescents using telehealth services reported effective communication with the provider compared with patients using in-person visits.

**Conclusions:**

In most studies, telehealth services received more favorable or comparable satisfaction ratings than in-person visits. Needed improvements in telehealth services included strategies to address technological challenges and develop better rapport among the patient, caregiver, and medical provider. Interventions may investigate the influence of telehealth services on access to and quality of care.

## Introduction

### Background

Many health care providers switched from using in-person to remote care for medical visits to adhere to federal, state, and local stay-at-home orders during the COVID-19 pandemic. A 154% increase in the use of telehealth services occurred in March 2020 compared with March 2019 [1]. Telehealth is defined as “the remote provision of health care services through telecommunication technologies for prevention, diagnosis, and treatment” [2].

Telehealth can be synchronous care, asynchronous care, and remote monitoring [3]. Synchronous care entails a direct conversation between the patient and health care provider using telecommunication technology to complete a health care appointment. Asynchronous telehealth involves patient-provider interactions via email, text messaging, or patient portals, where medical questions, photographs, test results, reminders, and medical history are exchanged [3]. Remote monitoring can include receiving frequent vital signs and photographs from patients for detection and intervention [4].

The use of telehealth services has limitations. Previous studies indicated that technological issues [5], incomplete appointments [5], the lack of in-person and personable interaction [5-7], linguistic barriers to access [6], and lower compassion in care [5] were areas of concern in the use of telehealth services. To improve these downfalls in telehealth care, understanding satisfaction with pediatric telehealth services compared with pediatric nontelehealth services provides insight into health care enhancements.

The literature surrounding the use of telehealth services during the COVID-19 crisis is growing, but to the best of our knowledge, no systematic review assessed satisfaction with pediatric care comparing separate groups of participants, where at least one group received telehealth services and another group received nontelehealth services during the time frame of the pandemic. In one published scoping review focusing on both adult and pediatric care during the COVID-19 pandemic, satisfaction was mentioned briefly, stating that there was insufficient information on that topic and proposed researching satisfaction in the future [7]. In a systematic literature review of pediatric randomized controlled trials (RCTs) conducted before the COVID-19 pandemic, telemedicine interventions resulted in slightly better or comparable satisfaction with care and health outcomes compared with control groups in all 10 included studies [8].

### Objective

Our study sought to investigate satisfaction with telehealth during the COVID-19 pandemic compared with nontelehealth use among caregivers and pediatric patients. Our systematic literature review included only studies that compared satisfaction in separate groups of participants (ie, caregivers and pediatric patients), where at least one group participated in telehealth services and 1 group received nontelehealth services. Because of stay-at-home orders, studies with comparison groups for nontelehealth services that took place either before or during the pandemic were eligible.

## Methods

### Research Question, Review Design, and Eligibility Criteria

#### Overview

The review question was as follows: what is the satisfaction of pediatric patients and their caregivers involved in telehealth compared with those involved in nontelehealth services? We followed the 2020 PRISMA (Preferred Reporting Items for Systematic Reviews and Meta-Analyses) guidelines [9]. The page numbers where different elements of the PRISMA are included in this study are in the completed PRISMA 2020 checklist in Multimedia Appendix 1. Before conducting the literature search, the review protocol was registered in the International Platform of Registered Systematic Review and Meta-analysis Protocols (INPLASY; registration 202290067) [10].

One of the PRISMA 2020 checklist items is “Describe and explain any amendments to information provided at registration or in the protocol” [9]. In the original registered review protocol, the intent was to not restrict to only studies with comparison groups. Owing to the large number of studies with no comparison groups (ie, 187 studies), we restricted our search to only studies with comparison groups. Initially, the study included pediatricians along with pediatric patients and caregivers as participants; as we were reviewing studies, we realized that the same pediatricians were asked to compare their in-person and telehealth visits without separating medical providers into comparison groups. In addition, our search was initially conducted without the guidance of a librarian, resulting in 13 eligible studies. As a manuscript focusing on how to conduct literature searches following PRISMA guidelines states, guidance provided by librarians can result in reproducible searches [11]. Revising the search strings through the collaboration of a librarian resulted in 1 additional article or a total of 14 studies. Thus, the review protocol was revised to include a comparator, exclude pediatricians as part of the participants (ie, exclude pediatricians describing their satisfaction and only include pediatric patients and their caregivers), and revise the search strategy by using Medical Subject Headings (MeSH) words as described below based on the suggestions of a librarian [10]. The Populations, Interventions (or Exposures), Comparators, Outcomes, and Study designs or Settings (PI(E)COS) structure used in this review is as follows:

- Outcome: satisfaction
- Participants: pediatric patients and their caregivers
- Intervention or exposure: telehealth
- Comparison group: a group not participating in telehealth services, such as a group receiving in-person services
- Time frame: COVID-19 pandemic

#### Language and Study Designs

The inclusion criteria were peer-reviewed studies with full text in the English language seeking to gain perspectives on satisfaction among pediatric patients and their caregivers involved in telehealth services compared with pediatric patients and their caregivers involved in nontelehealth services. Conference abstracts and dissertations were also excluded.

There were no restrictions on the study design because of the limited number of studies with comparison groups. The 2020 PRISMA guidelines state that checklist items “are applicable to systematic reviews with objectives other than evaluating interventions” [9]. All intervention designs were included specifically randomized RCTs, quasi-experimental studies with control or no control groups, and qualitative studies. In addition to interventions studies, observational cross-sectional, cohort, and case-control studies involving surveys and interviews assessing satisfaction among those participating in telehealth services compared with those participating in nontelehealth services were also included in the study. Both prospective and retrospective analyses were eligible for inclusion in the study.

#### Health Conditions

Pediatric patients aged between 0 and 18 years could be seen for any physical or mental health condition in this review. No restrictions on the type of condition were placed. Studies on satisfaction during pregnancy were also excluded.

#### Time Frame

Telehealth services that occurred during the COVID-19 pandemic were also included. As at times, only telehealth services may have been allowed because of stay-at-home orders, studies with comparison groups for nontelehealth services that took place either during or before the pandemic were included. As search words for the COVID-19 pandemic were used, the search was not restricted by date. Interventions and observational studies with published dates occurring after the onset of the pandemic were excluded if they did not address the implications of the COVID-19 crisis or if the study period occurred before the pandemic. Studies from the period when the COVID-19 crisis had not yet been defined as a pandemic were excluded.

#### Participants

Patients (ie, children and adolescents aged 0-18 years) and caregivers (eg, family members, parents, mothers, or fathers) were included. Studies in which adults discussed experiences with their own health care and nonpediatric care were excluded.

#### Intervention or Exposure

Studies in which participants received telehealth services such as video and telephone visits and remote monitoring were included. No restrictions were imposed on the type of telehealth.

#### Intervention or Exposure Comparator

Studies in which there was at least one comparison group of participants who were receiving nontelehealth services, such as in-person visits, were included. Studies comparing telehealth services and nontelehealth services in the same group of participants (such as in studies where the same patients were asked to compare their experiences with telehealth services and nontelehealth services) were excluded.

#### Outcome

No standard measurement of satisfaction was included in the study. Studies that created their own measurements, as well as studies that used reliable or valid or other measurements, were included.

#### Databases and Search Strategy

Literature review searches were performed in the PubMed, CINAHL, Embase, and PsycINFO databases on January 5, 2023. The search strings were developed with the guidance of a librarian from the University of Michigan—Flint library, as stated in the *Acknowledgments* section. The search strings in Multimedia Appendix 2 were used in each database. Multimedia Appendix 2 also lists the specific steps of the search, such as a description of whether an advanced search was used and in what box or field the strings were entered so that one could reproduce the searches.

As different countries may use different synonyms for keywords, we sought to use various synonyms. Synonyms for exposure were “telehealth,” “telemedicine,” “video consultation,” and “remote consultation.” Synonyms of the outcomes were “patient satisfaction,” “satisfaction,” “perception,” and “attitude.” Synonyms for the type of care and population included “pediatrics,” “paediatric,” “pediatric,” “baby,” “infant,” “child,” “teen,” and “adolescent.” Synonyms for the time frame were “Covid-19,” “SARS-CoV-2,” “sars-cov-2,” “sars-cov-2,” “Severe Acute Respiratory Syndrome Coronavirus,” “NCOV,” “2019 NCOV,” and “coronavirus.”

PubMed and Embase allowed for the use of MeSH and explosion (marked by /exp) terms, respectively, which used their own synonyms as part of the search. In PubMed, the following MeSH terms were entered: “pediatrics,” “child,” “infant,” “adolescent,” “telemedicine,” “remote consultation,” “patient satisfaction,” “COVID-19,” “sars-cov-2,” and “coronavirus.” Telemedicine is a MeSH term introduced in 1993 that is associated with the following synonyms: “telehealth,” “tele-referral,” “virtual medicine,” “tele-intensive care,” “tele-ICU,” “mobile health,” “mHealth,” and “eHealth.”

Essential keywords were entered both as MeSH words or expanders and separately with truncations; therefore, the search allowed the inclusion of more relevant articles. Specifically, “pediatric,” “paediatric,” “child,” “adolescent,” “attitude,” and “perception” were entered as MeSH/explosion terms and separate words with truncations. The symbol for truncation is the asterisk (*), which allows for the retrieval of all words that contain the part of the term preceding the asterisk. Truncations in all databases were as follows: “pediatric*,” “paediatric*,” “child*,” “adolescen*,” “attitude*,” and “perception*.” MeSH words and expanders must be entered without truncations.

### Data Extraction and Synthesis

After using the search words in Multimedia Appendix 2 in each database, all articles were uploaded to EndNote Basic (Clarivate), where duplicates were removed using automation tools. Following the removal of duplicates, 2 authors (GDK and TC) reviewed all the remaining titles and abstracts. If the title and abstract did not clearly indicate whether the study met or did not meet the inclusion criteria, the reviewer opened the full text of the article. Each reviewer made notes about why the study fit the inclusion or exclusion criteria in a separate Excel file. The 2 reviewers resolved disagreements after reviewing the full text of the article. A consensus was reached between the reviewers on the items when there were differing opinions on inclusion in the literature review.

The final studies were categorized based on the participant group whose satisfaction was assessed. Information was extracted on the location, sample size by comparison group, mean and median age of participants or other information provided on age, pediatric health condition, telehealth and comparison group definitions, assessment of satisfaction, and findings by comparison group. Some studies used the term “telehealth” and others used “telemedicine.” The terms referred to in these studies were used.

If the article did not specify the type of study design used, we made decisions on what the study design was based on descriptions in the text. Owing to the many satisfaction findings in most studies, not all outcomes were extracted in tables. Findings representative of the overall study results were extracted; for example, for studies that concluded that patients were more satisfied with in-person visits than telehealth visits, representative findings showing lower satisfaction with telehealth visits were extracted. If a study used both close-ended and open-ended items, the key results for both types of items were presented. Findings extracted were not based on the same measures because the studies used various definitions of satisfaction. Risk ratios, mean differences, and P values, if any, were reported as the key results. GDK conducted data extraction. TC reviewed the extracted data. GDK and TC reviewed the final data and resolved any disagreements through discussions.

### Quality of Evidence or Risk of Bias

The quality of evidence in the studies was independently evaluated by 2 reviewers (GDK and TC) using 2 methods: the Grading of Recommendations, Assessment, Development, and Evaluation (GRADE) approach [12] and the Joanna Briggs Institute (JBI) critical appraisal tools [13].

The GRADE approach involves assigning a quality level rating to a study based on the study design [12]. According to the GRADE approach, RCTs receive a high rating, and observational studies receive a low rating [12]. In this review, the ratings based on study design were as follows: RCTs, high; quasi-experimental pretest and posttest study, moderate; cohort study (prospective) and case-control study (retrospective), low to moderate; and analytic cross-sectional study (retrospective), low. Similarities and differences in sociodemographic characteristics of participants in comparison groups and validity and reliability of data collection tools were reviewed in more detail in a narrative table to assess limitations in the study design and execution of the study in Multimedia Appendix 3 [14-27].

In addition, to assess specific limitations in the study design and execution of the studies, scores were assigned based on answering common questions across study types on JBI forms [13] in Multimedia Appendix 4 [14-27]. In total, 2 independent reviewers (GDK and TC) used the forms separately with JBI assessment form questions for each study including the following:

- Were the criteria for inclusion in the sample clearly defined?
- Were the study participants and setting described in detail?
- Were the comparison groups (telehealth and nontelehealth) comparable in terms of sociodemographic characteristics?
- Was the period (such as months and years) of the study periods for each comparison group clearly defined?
- Was satisfaction measured in the same way in the comparison groups?
- Were satisfaction outcome measures valid and reliable?
- Were appropriate statistical analyses used?

A score of 1 for the above questions meant “yes,” a score of 0.5 signified “partially,” and a score of 0 meant “no or unclear.” The scores were summed up for each question for each study. Higher total scores indicated higher study quality and a lower risk of bias. Disagreements in GRADE quality levels and JBI critical appraisal forms scores were resolved through discussion between the 2 reviewers.

## Results

### Literature Search

**Figure 1:**
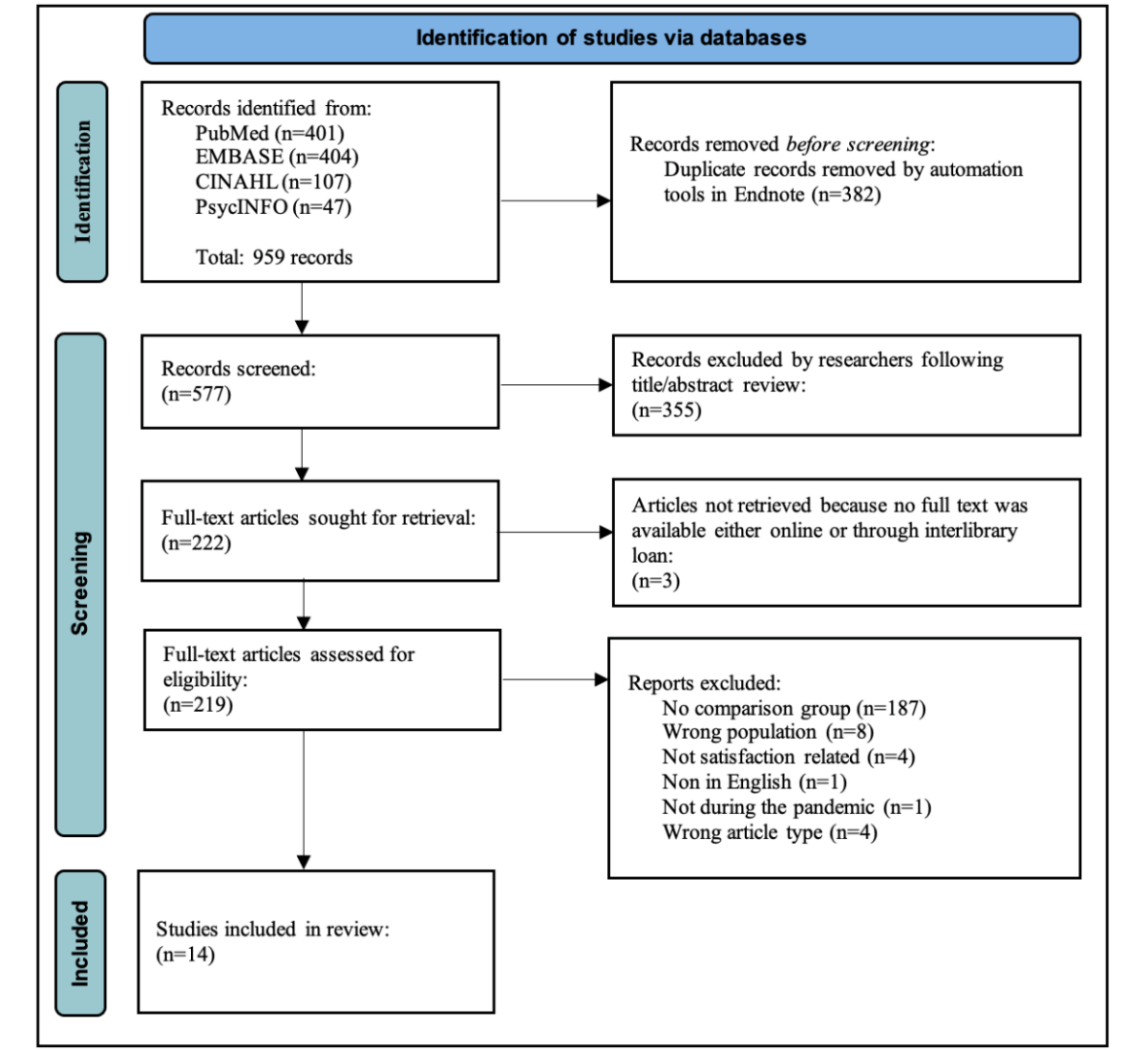
Identification of studies through the systematic review process.

Figure 1 illustrates the flow of the systematic review in selecting articles. A total of 14 manuscripts met the inclusion criteria [14-27].

Of the 959 articles following the original search, 382 (39.8%) were removed using automation tools because they were duplicates. A total of 355 articles were removed during title or abstract review. Among the 219 full-text articles assessed, 187 (85.3%) were excluded because they focused on satisfaction in pediatric care during the pandemic but did not have a comparison group. An example of a study that was excluded was that from Zambia, in which 1 group of adolescents answered questions about experiences and perceptions related to telephone visits without comparing the results with a group of adolescents having nontelehealth visits [28]. Of the 187 articles, only 14 (7.4%) assessed satisfaction with health care among pediatric patients and their caregivers participating in telehealth services compared with those participating in nontelehealth services.

Among the 14 studies [14-27], 13 (93%) assessed satisfaction among caregivers by asking them about the health care of their children [14-26] and 1 (7%) [27] assessed satisfaction by asking caregivers and adolescents (ie, patients) about their satisfaction separately. Furthermore, 1 study compared video, in-person, and hybrid visits [14], and 1 study compared telephone visits with in-person visits [25]. Another study compared video, telephone, and in-person visits [24]. All others compared video and in-person visits.

Table 1 provides the location, number of participants, number of visits, mean and median age of participants or other information provided on age, and the pediatric health condition within each study. Table 2 includes information on the telehealth and comparison group definitions, assessment of satisfaction, and findings for telehealth and nontelehealth groups.

**Table 1:** Location, number, age, and condition among participants in the 14 included studies.

Study			Country		Number of participants or number of visits		Mean or median age of participants or other information provided on age		Pediatric health condition
**Caregivers or patients: caregivers completed survey items on behalf of children or adolescents; results for caregivers and patients were presented together without presenting separate results for patients**									
	Corona et al [14], 2021	United States		Total: 95 patients Video visit patients: 46 In-person visit patients: 49		Video visits patient mean age: 28.17 months In-person visits patient mean age: 27.96 months		Autism spectrum disorder	
	Hoi et al [15], 2022	United States		Total: 21,592 visits Telemedicine visits: 2051 In-person visits: 19,541		Telemedicine visits patient mean age: 7.2 years In-person visits patient mean age: 7.3 years		Otolaryngology	
	Holzman et al [16], 2021	United States		Total: 153 patients Telemedicine visit patients: 51 In-person visit patients: 102		Telemedicine visits patient median age: 8.0 years In-person visits patient median age: 7.5 years		Urology	
	Johnson et al [17], 2020	United States		Total: 15,562 visits Telemedicine visits: 11,192 In-person visits: 4370		Not stated		A variety of pediatric specialties, such as attention-deficit hyperactivity disorder, adolescent health, developmental and behavioral, cystic fibrosis	
	Katz et al [18], 2021	United States		Total: 150 patients Telemedicine visit patients: 23 In-person patients: 127		Telemedicine visits patient age distribution: <1: 0% 1-4: 17.39%, 5-10: 39.13% 11-14: 30.43% ≥15: 13.04% In-person visit patient age distribution: <1: 29.13% 1-4: 33.07% 5-10: 25.2% 11-14: 8.67% ≥15: 3.94%		Primary care	
	Kennelly et al [19], 2021	United States		Total: 1514 visits Telemedicine visits: 688 In-person visits: 826		Not stated		Development pediatrics and autism	
	Love et al [20], 2022	United States		Total: 232 Telemedicine visit patients: 156 In-person visit patients: 76		Telemedicine visits patient age distribution: <1: 6% 1-10: 44% ≥10: 50% In-person visit patient age distribution: <1: 1% 1-10: 51% ≥10: 47%		Pediatric gastroenterology	
	Mahmoud et al [21], 2022	Egypt		Total: 1928 patients Telemedicine visit patients: 1056 In-person visit patients: 874		Telemedicine visits patient mean age: 1.2 In-person visits patient mean age: 1.6		Ambulatory surgery	
	Marques et al [22], 2020	United States		Total: 9392 visits Telemedicine visits: 2960 In-person visits: 6362		Not stated		Ambulatory care	
	McCoy et al [23], 2022	United States		Total: 172 patients Telemedicine visit patients: 59 In-person visit patients: 113		Telemedicine visits patient mean age: 4.99 In-person visits patient mean age: 6.15		Otolaryngology	
	Mustafa et al [24], 2021	United States		Total: 401 patients Video visit patients: 98 In-person visit patients: 303		Video visits patient mean age: 29 (range 11-52.5) years In-person visits patient mean age: 20 (range 6-52.5) years		Allergy or immunology	
	Ragamin et al, 2021 [25]	The Netherlands		Total: 144 patients		Patient median age: 6 years		Atopic dermatitis	
	Summers et al [26], 2022	United States		Total: 817 patients Video visit patients: 674 In-person visit patients: 143		Not stated		Ophthalmology	
**Satisfaction assessed separately for adolescents and caregivers: adolescents answered at least some satisfaction surveys items separately; results were presented separately for adolescents and caregivers**									
	Troncone et al [27], 2022	Italy		Total: 610 patients Video visit patients: 305 In-person visit patients: 305 Video visit caregivers: 305 In-person visit caregivers: 305		Video visits patient mean age: 12.17 years In-person visits patient mean age: 12.12 years		Type 1 diabetes	

**Table 2:** Telehealth approach, assessment of satisfaction, and findings among the 14 included studies.

Study		Telehealth approach and comparison groups	Satisfaction data collection tool examples	Findings
**Caregivers or patients: caregivers completed survey items on behalf of children or adolescents; results for caregivers and patients were presented together without presenting separate results for patients.**				
	Corona et al [14], 2021	Video sessions between March 2020 and August 2020 compared with in-person visits between July 2019 and March 2020	A 14-item closed-ended survey developed by the authors, including items, such as “I would recommend these services to other families,” “My child’s behavior and skills improved during this service,” and “I am pleased with the outcome of services for me and my child.”	89% of caregivers participating in telehealth service strongly agreed that they were pleased with the outcome of the visit compared with 87% of caregivers participating in in-person visits.
	Hoi et al [15], 2022	Telemedicine visits consisting of 352 new patient video visits, 1548 return video visits, 109 phone visits, 41 nonportal video visits for caregiver without portal access, and 1 patient education video visit compared with in-person visits consisting of new patient visits, return visits, preoperative visits, and postoperative visits between April 1, 2020, and April 30, 2021	Surveys with closed-ended and open-ended items. Surveys asked respondents to express a positive or negative sentiment with the visit as well as to provide narrative comments on the experience with the visit.	96% of caregivers or patients reported positive experiences with the in-person visit compared with 100% with the telemedicine visit. An example of a positive comment regarding the in-person visit was, “All of my concerns about my son’s health have been listened to.” An example of a positive comment regarding the in-person visit was, “The video visit saved us a 10-h drive.”
	Holzman et al [16], 2021	Video visits between April 2020 till 2020 compared with in-person visits between January 2019 and March 2020	Survey with a close-ended question on satisfaction: “using a number from 1 to 10, where 1 is the worst provider possible and 10 is the best provider possible, what number would you use to rate this provider?”	92% of caregivers and patients participating in telemedicine visits reported high satisfaction with the provider compared with 87% of caregivers and patients participating in in-person visits (odds ratio 1.7, 95% CI 0.53-5.7).
	Johnson et al [17], 2020	Video visits compared with in-person visits between April 2020 and May 2020	Survey with a closed-ended question on satisfaction: “using a number from 1 to 10, where 1 is the worst provider possible and 10 is the best provider possible, what number would you use to rate this provider?”	97.9% of caregivers and patients participating in telemedicine visits reported high satisfaction with the provider compared with 83.9% of caregivers and patients participating in in-person visits (P=.07).
	Katz et al [18], 2021	Video visits compared with in-person visits between March 10 and June 29, 2020	Survey with closed-ended questions on satisfaction, such as “Provider explained problem or condition” and “Provider made efforts to include us in decisions”	In 91.25% of telemedicine visits, caregivers indicated the provider provided a very good explanation of the problem or condition compared with 84.57% of in-person visits. This difference was not statistically significant. The mean visit satisfaction scores were 92.25% for in-person visits and 95.37 for telemedicine visits.
	Kennelly et al [19], 2021	Telemedicine visits for caregivers between June 2020 till July 2021 compared with in-person visits for caregivers/patients between June 2019 and May 2020	Press Ganey survey consisting of 21 items; 7 items not relating to telemedicine were excluded. A closed-ended item on overall assessment of care included likelihood of your recommending our practice to others.	87.25% of caregivers and patients participating in telemedicine visits were likely to recommend practice to others compared with 85.76% of caregivers and patients participating in in-person visits (P<.99).
	Love et al [20], 2022	In-person visits compared with video visits between May 2020 and June 2020	Oral telephone survey with questions 2 weeks after the visit on reasons for preferring the type of visit. The specific questions asked were not included. It appeared from the descriptions that the questions were closed ended.	Among those in the telemedicine group, the reasons for preferring the visit were time savings associated with less driving and reduced cost. Among those in the in-person group, the reasons for favoring the visit were having a preferred physician and wanting a physical examination.
	Mahmoud et al [21], 2022	Video visits between April 2020 till May 2020 compared with in-person visit between January 2020 and February 2020	Patient experience assessment survey with closed-ended items, such as “overall the service was excellent and it met my expectations.” Caregivers were asked for the reasons, if any, why they were dissatisfied with the visit experience.	92% of caregivers and patients participating in telemedicine visits were satisfied with the visit compared with 63% of caregivers and patients participating in in-person visits (P=.04). 8% of all caregivers and patients participating in telemedicine visits were dissatisfied with the visit because of not being persuaded by the video visit, having internet problems, and having a time that interfered with their schedule. 37% of all caregivers and patients participating in in-person visits were dissatisfied with the visit because of issues related to parking, cleanliness, wait time, and provider and reception office attitudes.
	Marques et al [22], 2022	In-person visits compared with video visits between January 2020 and December 2020	A 16-item closed-ended patient experience survey with items, such as how well the nurse listened to you; likelihood of recommending the practice to others; our concern for your privacy. Each question had ratings of 1-5. A rating of 5/5 was considered “top box.”	No statistically significant differences in satisfaction (as assessed by top box percentages) existed between caregivers participating in in-person and those participating in telemedicine visits.
	McCoy et al [23], 2022	Video visits for the first 6 weeks when telemedicine was implemented compared with in-person visits for the 6 weeks before telemedicine was implemented	Closed-ended items, such as: ”How would you rate the following aspects of your child’s experience:” ability to communicate with the physician and the overall outpatient experience.	The ability to communicate with the physician was rated higher (mean 4.6) in the in-person group compared with the telemedicine group (mean 4.4; P<.01)
	Mustafa et al [24], 2021	Video new and follow-up visits compared with in-person new and follow-up visits from June 26, 2020, to July 31, 2020	Closed-ended items, such as, “Overall, I was satisfied with my in-person/video encounter; my in-person/video encounter resulted in a complete evaluation; in the future, I would prefer the following visit type: in-person, video etc.” Open-ended items, such as, “What is the most important reason you would prefer an in-person encounter?”	81.5% responded that they strongly agreed that they were satisfied with the in-person visit compared with 72.7% with the video visit. The most frequently mentioned reason caregivers were satisfied with an in-person visit was to have a physical examination. Another theme was “in-person care allows for a more personal interaction and more questions.” Reasons why respondents were satisfied with video visits included: frequent follow-up, convenience, and COVID-19 safety.
	Ragamin et al [25], 2021	New and follow-up in-person consultations compared with new and follow-up telephone consultations from March 2020 to July 2020. Telephone consultations could be with or without shared imagines via email to aid the visit.	Closed-ended items, such as, “What is your general satisfaction with received care?” and “Assess your patient satisfaction in these areas: information provided, active involvement, needs addressed, emotional support, interaction in general” Open-ended items on reasons why caregivers preferred face-to-face or remote consultations.	34.7% of caregivers who received face-to-face consultations were very satisfied compared with 12.1% of caregivers who received remote consultations (P<.001). Caregivers who received face-to-face consultations were significantly more satisfied on the emotional support scale compared with those who received remote consultations (P=.039). Reasons why caregivers preferred face-to-face consultations included “face-to-face examination is important”; “face-to-face consultations raise treatment adherence”; “face-to-face consultations are more efficient.”
	Summers et al [26], 2022	In-person visits compared with telehealth visits completed by caregivers and patients between March 2020 and July 2020	Patient satisfaction survey administered by an outside company with items, such as: “How likely would you be to recommend this facility to your family and friends?” with a score of 0 being not all likely and 10 being extremely likely.	The satisfaction survey scores for “recommend institution” and “recommend provider” were 79.6 and 86.4, respectively, for in-person visits and 81.4 and 92.3, respectively, for telehealth visits.
**Satisfaction assessed separately for adolescents and caregivers: adolescents answered at least some satisfaction surveys items separately; results were presented separately for adolescents and caregivers.**				
	Troncone et al [27], 2022	Video visits between April 2020 and May 2020 compared with in-person visits between June 2020 and July 2020	Patients themselves completed the JSPPPE^a^. Caregivers completed the CASC^b^. Examples of CASC items included “I would like to see improvement in the comfort and support the provider gave to the child: yes/no.”	Adolescents: the mean JSPPPE score was 28.92 for video consultations and 27.82 for the in-person visits showing slightly higher satisfaction with the video visit, which was not statistically significant (P=.096). Caregivers: there were no statistically significant differences in responses to items as part of CASC. For example, 83% of caregivers participating in video visits answered no to, “I would like to see improvement in the comfort and support the provider gave to the child” compared with 81% of caregivers participating in in-person visits (P=.597).

^a^JSPPPE: Jefferson Scale of Patient Perceptions of Physician Empathy.

^b^CASC: comprehensive assessment of satisfaction with care.

### Study Characteristics

In total, 11 studies were conducted in the United States [14-20,22-24,26] and 1 each was conducted in Egypt [21], the Netherlands [25], and Italy [27] (Table 1). Some studies provided information only on the number of visits and other studies provided information on the number of participants. The sample sizes of the participants ranged from 23 to 1056 telehealth visit patients and from 49 to 874 in-person visit patients.

Table 2 includes the periods for telehealth and nontelehealth services. In all 14 studies, telehealth services were provided during the pandemic. In 5 of the 14 studies [14,16,19,21,23], nontelehealth services were provided before the pandemic, and in the remaining 9 investigations, nontelehealth services were provided during the pandemic. In the 5 studies where nontelehealth services were provided before the pandemic, satisfaction was higher with remote compared with in-person visits in 4 studies [14,16,19,21], whereas it was higher with in-person than remote visits in 1 study [23].

### Quality and Risk of Bias Assessment

#### Overview

Multimedia Appendix 3 includes information related to quality including study design, quality of evidence rating, similarities and differences in sociodemographic characteristics among the comparison groups, and validity and reliability of the data collection tools. Multimedia Appendix 4 includes the risk of bias scores based on the common questions of the JBI critical assessment tools.

#### Study Design

Among the 14 studies, there was 1 intervention: a pretest-posttest 3-group intervention [14] (Multimedia Appendix 3). In the pretest-posttest 3-group intervention, the 3 comparison groups were families who received behavioral and support sessions through telemedicine only, families who received sessions through in-person interaction only, and families who received the intervention in a hybrid mode [14].

Of the 14 studies, 5 were prospective cohort investigations [15,20,23,24,27]. In these prospective cohort studies, investigators classified individuals as participating in nontelehealth or telehealth services and then followed them over time to assess their satisfaction. In addition, 1 study was a retrospective case-control investigation that used propensity score matching to match individuals who had in-person and telehealth services in the past [16]. Furthermore, 7 studies used retrospective analyses in which answers to satisfaction surveys between individuals participating in telehealth and nontelehealth services were compared at certain periods in the past [17-19,21,22,25,26]. On the basis of the types of design according to the GRADE approach, the distribution of gradings were 7 studies with a low grading, 6 studies with a low to moderate grading, and 1 study with a moderate grading.

#### Similarities and Differences in Participants Between the Nontelehealth and Telehealth Groups

In 6 studies, there were no sociodemographic comparisons of participants between telehealth and nontelehealth groups [17,19,20,22,25,26] (Multimedia Appendix 3). In most studies that reported sociodemographic characteristics of telehealth and nontelehealth groups, there were both similarities and differences between the groups. For example, in 1 study, the 2 groups (telehealth and in-person) were similar in terms of race and ethnicity (94.7% of White participants in the in-person group and 94.9% of White participants in the telehealth group) but not in terms of sex (61.9% of male participants in the in-person group and 49.2% of male participants in the telehealth group) [23]. In another study, the 2 groups (telehealth and in-person) were similar in terms of sex but not in terms of residence (66.4% of participants in the remote care group were rural residents compared with 47.7% of participants in the in-person group) [21]. In a study with propensity score matching to separate patients seen in the past into 2 groups, the telehealth and in-person groups were similar in terms of age, language, and type of visit but not in terms of sex [16].

#### Outcome Variable (ie, Satisfaction) Reliability and Validity

Some authors have used reliable and valid surveys (Multimedia Appendix 3), such as the Jefferson scale of patient perceptions of physician empathy (Cronbach α=.896) [26]. A total of 8 studies provided no information regarding the reliability and validity of the satisfaction survey [14,15,17,20,21,23,24,26]. In 1 study, the authors specified that they developed their own satisfaction survey [14]. Satisfaction was measured in the same way in all studies, except for in 1 [20], in the telehealth and nontelehealth groups.

#### Quality of Evidence Scores Based on JBI Critical Assessment Tool Common Questions

The total scores among the 14 studies are included in Multimedia Appendix 4. The reasons for decreased scores included limited description of the participants and settings, differences in sociodemographic characteristics between participants in the comparison groups, the lack of valid and reliable measures of satisfaction, and not using the same questions for participants in the telehealth and nontelehealth groups.

### Satisfaction Among Caregivers Completing Survey Items on Behalf of Children and Adolescents

In 13 studies focusing on caregiver satisfaction [14-26], caregivers completed the survey items (Table 2). The results for caregivers and patients were presented together without presenting separate results based on the patient perspective in the 13 studies. Examples of close-ended survey items to assess satisfaction were as follows: “My child’s behavior and skills improved during this service” [14], “I am pleased with the outcome of services for me and my child” [14], “Overall the service was excellent and it met my expectations” [21], and “How would you rate your child’s ability to communicate with the physician?” [23]. Of the 13 studies, 4 involved the use of open-ended items in addition to close-ended items to assess satisfaction in surveys [15,21,24,25]. Examples of open-ended questions as part of the surveys (not interviews) were as follows: “Provide narrative comments on your experience with the visit” [15], “What were the reasons you were dissatisfied with the visit experience?” [21], and “What is the most important reason you would prefer an in-person/telehealth encounter?” [24,25].

In 8 of the 13 studies on caregivers, participants in telehealth services were more satisfied with care compared with participants in nontelehealth services. For example, in the study of Holtzman et al [16], 92% of caregivers and patients participating in telemedicine visits reported high satisfaction with the provider compared with 87% of caregivers and patients participating in in-person visits (odds ratio 1.7, 95% CI 0.53-5.7). In the study of Johnson et al [17], 97.9% of caregivers and patients participating in telemedicine visits reported high satisfaction with the provider compared with 83.9% of caregivers and patients participating in in-person visits (P=.07).

In 1 out of the 13 studies focusing on caregivers, satisfaction levels in people receiving telehealth services and those receiving in-person visits were very similar. Specifically, no statistically significant differences in satisfaction (as assessed by top box percentages) existed between in-person caregivers and those participating in telemedicine visits [22].

In 4 of the 13 studies focusing on caregivers, satisfaction tended to be higher with in-person visits compared with telehealth visits. For example, 34.7% of caregivers who received in-person consultations were very satisfied compared with 12.1% of caregivers who received remote consultations (P<.001) [25]. In a study by Mustafa et al [24], 81.5% responded that they strongly agreed that they were satisfied with the in-person visit compared with 72.7% with the video visit. In a study by McCoy et al [23], the ability to communicate with the physician was rated higher (mean 4.6, SD 0.8) in the in-person group compared with the telemedicine group (mean 4.4, SD 0.7; P=.01).

Open-ended items offered more specific information on why the participants were satisfied with the visit. The strengths of the visits indicated by caregivers using telehealth included time savings [15], convenience [24], frequent follow-up [24], and protection from COVID-19 [24]. The limitations indicated by caregivers using telehealth included poor technological access and connectivity [21], limited personal connection with providers [24], and the lack of laboratory testing and physical examination [24]. The strengths of nontelehealth services indicated by caregivers using in-person visits included being listened to [15], having opportunities for personal interactions and questions [24], and ensuring treatment adherence [25].

### Satisfaction Among Patients and Caregivers

Overall, both pediatric patients and caregivers participating in telehealth appointments in separate surveys noted higher satisfaction when talking to their provider than patients and caregivers having in-person visits, although these findings were not statistically significant [27]. Specifically, among adolescents, the mean satisfaction score was 28.92 for video consultations and 27.82 for the in-person visits showing slightly higher satisfaction with the video visit, which was not statistically significant (P=.096) [27]. In the same study, there were no statistically significant differences in responses to items as part of the caregiver satisfaction survey. For example, 83% of caregivers participating in video visits answered no to “I would like to see improvement in the comfort and support the provider gave to the child” compared with 81% of caregivers participating in in-person visits (P=.597) [27].

## Discussion

### Principal Findings

The 14 included studies focused on satisfaction among caregivers and patients in a variety of pediatric care needs and specialties, including allergy and immunology, developmental and behavioral health conditions, concussion, type 1 diabetes, otolaryngology, ophthalmology, urology, gastroenterology, primary care, and ambulatory care. This literature review focused only on studies in which there was at least 1 comparison group of participants (ie, pediatric patients and caregivers) for telehealth and 1 comparison group of participants for nontelehealth services. Although the literature search resulted in 187 studies focusing on satisfaction with pediatric telehealth in general, only 14 studies included a comparison group for nontelehealth services. A reason for the limited number of articles with comparison groups may be that there were periods during the pandemic when patients relied only or mostly on remote care for their health care needs.

Most studies compared video and in-person visits. Only 1 of the 14 studies was an intervention (pretest-posttest quasi-experimental with 3 comparison groups) [14]. Previous literature reviews did not focus on comparison groups of telehealth and nontelehealth services in pediatric care [29-32].

In this review, a trend of overall higher or comparable satisfaction with telehealth compared with in-person visits in a pediatric setting was observed in most studies. Open-ended items were especially helpful in understanding the reasons for the satisfaction ratings. High satisfaction ratings with telehealth were commonly because of convenience and health benefits. The benefits of telehealth included not requiring transportation, ease of use, ability for frequent follow-up, and reduced likelihood of contracting the COVID-19 virus.

Only 1 study in which adolescents completed their own surveys on satisfaction was included in this review. The study found higher satisfaction with video visits compared with in-person visits among adolescents, although the difference was not statistically significant [27]. In an investigation conducted before the pandemic, adolescents and their caregivers were randomly assigned to an in-person visit and a video visit [33]. The mean for positivity for the telehealth visit was slightly higher at 5.53 compared with 5.37 for the in-person visit among adolescents [33].

One finding in this review was the concern for the lack of physical examination of patients during telehealth visits. In 1 study included in this review, 48% of caregivers having remote visits noted that the lack of physical examination was the greatest limitation of telehealth services [20]. Therefore, the ability to undergo physical examination is vital to the participants. Home-monitoring equipment may supplement remote appointments and prevent misdiagnoses [34]. To make monitoring equipment available and equitable, health care entities may offer these devices as loans for patients to use during remote appointments through mail-in programs, thus reducing disparities in telehealth use among patients.

Another key finding was the concern that telehealth appointments were less personalized compared with in-person appointments. For example, in 1 reviewed study, a theme based on responses to open-ended items was that in-person visits offered opportunities for personal interaction and asking more questions [24]. In another study, caregivers who received in-person consultations were significantly more satisfied with the emotional support scale compared with those who received remote consultations (P=.039) [25]. A prior study that asked open-ended questions of 105 caregivers similarly found that a lack of in-person interaction was a challenge for telehealth use [35]. Caregivers stated that they lost the feeling that the provider was compassionate during remote care [35]. To increase empathetic care during telecare, health care providers may look straight at the camera; offer verbal cues when needing to look away; start appointments with mutual agenda and goal setting; avoid the use of medical terminology; ask how the health condition affects the daily life of the patient; express knowledge of the patient’s history by mentioning prior visits; and, if possible, suggest in-person visits to supplement remote appointments [36].

Studies have identified issues with technology during telehealth appointments. Quality and affordable internet access is essential, and a lack of technological services should not prevent pediatric patients from receiving the care they need. To improve satisfaction in this area, we propose continued support in making access to internet services and technological products more readily available and equitable to families of different racial and ethnic and socioeconomic groups. In the United States, a federal program plans to cover 85% of the costs associated with broadband connectivity and network equipment needed to support connected care in selected locations by allocating over US $100 million over 3 years through competitive funding processes [37].

### Limitations of Reviewed Studies

Limitations included a nonrandomized design, small sample size, low generalizability beyond a specific health care setting or group, sampling method (ie, convenience sampling), limited diversity in backgrounds or no information on sociodemographic characteristics among participants, and the lack of reliable and valid satisfaction assessment tools. Among studies that presented sociodemographic characteristics of participants, differences in the comparison groups may have affected the findings. For example, in 1 study, 66.4% of participants in the telehealth group were rural residents, compared with 47.7% in the in-person group [21]. Rural participants may be more likely to have favorable views toward telehealth compared with urban residents because of transportation barriers associated with attending in-person visits in remote locales.

Most studies assessed satisfaction related to videoconferencing in a telehealth setting. Few studies have assessed satisfaction with telephone appointments [24,25]. In a study comparing telephone and in-person visits, telephone consultations could be conducted with or without shared images via email to aid the visit [25]. Another limitation is that in some studies, video visits were conducted using different technologies, such as FaceTime and Skype [24,27]. Time constraints and lack of assessment of longitudinal changes were other limitations of the reviewed studies. Another limitation is that most studies did not survey adolescents on their own opinions.

### Limitations of This Review

Represented in this review were peer-reviewed articles in the English language only. More eligible articles may have been published after the search date. Only the studies in the databases that were searched were included in this review. Other synonyms for the main keywords may have yielded additional eligible results. The use of additional or shorter truncations in all the databases may have resulted in more eligible articles.

Lower income or developing countries may use other terms to describe telehealth not included within the umbrella of the MeSH or explosion terms. We located studies without comparison groups in countries classified as lower income or developing, specifically Argentina [38], Brazil [39,40], India [41-44], Jordan [45], North Macedonia [46], Philippines [47], Saudi Arabia [48,49], and Zambia [28], which used the terms telehealth, telemedicine, and remote consultation. Therefore, we believe that this review allowed for various countries to be included. Still, although the review did not place restrictions on country, 13 of the 14 included studies were from developed nations. It is possible that studies in developing nations did not have as many resources to conduct studies with comparison groups, which may be more costly. The results were mostly applicable to higher income countries because the characteristics of telehealth in lower income countries may be distinct.

Another limitation was the inclusion of different populations (such as by age and type of condition). Owing to stay-at-home orders, in some studies, nontelehealth services were conducted before the pandemic when telehealth services were not as widespread. Participants in comparison groups for nontelehealth services before and during the pandemic may have had different perceptions of in-person visits. Given the different designs and measurements among the studies, we did not perform a meta-analysis.

### Future Research

Much prior research has focused on satisfaction with telehealth services without including a comparison group for nontelehealth services [44,50-70]. Additional research comparing satisfaction with telehealth and nontelehealth pediatric services during the same timeframe will be beneficial. Such research provides insight into the type of support patients and caregivers need to access and use for telehealth and in-person visits. RCTs may compare the influence of telehealth and nontelehealth services on medical and pharmacy costs, the accuracy of the patient and caregiver recall, quality of health care, and pediatric health. In addition, comparing satisfaction with in-person laboratory visits and mobile laboratory visits is innovative.

Future studies involving focus groups and interviews with health care providers may offer insight into how telehealth and nontelehealth services influence a provider’s ability to conduct all the necessary examinations for a patient. Interventions may assess the influence of education for health care providers in strategies for personable interactions during telehealth appointments on patient and caregiver satisfaction with pediatric care. Education should provide information on how communication during telehealth visits can be tailored to the different cultural and linguistic groups of patients and caregivers.

### Conclusions

Telehealth visits were comparable or superior in terms of patient and caregiver satisfaction compared with in-person visits in most of the reviewed studies. This review identifies potential weaknesses of telehealth services that need improvement such as problems with technology connectivity, limited ability to undergo a physical examination, difficulty in having personable interactions with the medical provider, and lower adherence to treatment. Health care providers may develop strategies to overcome these weaknesses and improve telehealth during the pandemic and beyond.

## Appendix

Multimedia Appendix 1 PRISMA (Preferred Reporting Items for Systematic Reviews and Meta-Analyses) 2020 checklist.

Multimedia Appendix 2 Search strings by database.

Multimedia Appendix 3 Quality of evidence based on design, sociodemographic participant characteristics, and tool reliability and validity.

Multimedia Appendix 4 Quality of evidence scores based on Joanna Briggs Institute critical assessment tool common questions.

## Abbreviations

GRADE: Grading of Recommendations, Assessment, Development, and Evaluation
INPLASY: International Platform of Registered Systematic Review and Meta-analysis Protocols
JBI: Joanna Briggs Institute
MeSH: Medical Subject Headings
PI(E)COS: Populations, Interventions (or Exposures), Comparators, Outcomes, and Study designs or Settings
PRISMA: Preferred Reporting Items for Systematic Reviews and Meta-Analyses
RCT: randomized controlled trial
