# Supplementary material for: Satisfaction With Telehealth Services Compared With Nontelehealth Services Among Pediatric Patients and Their Caregivers: Systematic Review of the Literature
Source: JMIR Pediatr Parent. 2023 Apr 27;6:e41554. doi: 10.2196/41554 (PMC10176140; doi:10.2196/41554)
Supplement: Multimedia Appendix 2 [file pediatrics_v6i1e41554_app2.docx]

Multimedia Appendix 2: Search Strings by Database^

**PubMed**

**Steps: In** [**https://pubmed.ncbi.nlm.nih.gov/**](https://pubmed.ncbi.nlm.nih.gov/)**, select Advanced search, type the entire**

**search string into the Query box**

((((("Pediatrics"[Mesh]) OR ("Child"[Mesh]) OR ("Infant"[Mesh]) OR ("Adolescent"[Mesh]) OR (pediatric*[Title/Abstract]) OR (paediatric*[Title/Abstract]) OR (child*[Title/Abstract]) OR (infant[7yTitle/Abstract]) OR (baby[Title/Abstract]) OR (teen*[Title/Abstract]) OR (adolescen*[Title/Abstract]))) AND ((("Telemedicine"[Mesh]) OR ("Remote Consultation"[Mesh]) OR (telemedicine[Title/Abstract]) OR ("video consultation"[Title/Abstract])OR ("remote consultation"[Title/Abstract]) OR (telehealth[Title/Abstract]) OR ("remote consultation"[Title/Abstract])))) AND ("COVID-19" OR "COVID-19"[MeSH Terms] OR "SARS-CoV-2" OR "sars-cov-2"[MeSH Terms] OR "Severe Acute Respiratory Syndrome Coronavirus 2" OR "NCOV" OR "2019 NCOV" OR ("coronavirus"[MeSH Terms] OR "coronavirus" OR "COV"))) AND ((((("Patient Satisfaction"[Mesh]) OR (satisfaction[Title/Abstract])) OR (attitude*[Title/Abstract])) OR (attitude*[Title/Abstract])) OR (perception*[Title/Abstract]))

**EMBASE**

**Steps: In the subscription-only database available at**

[**https://www.embase.com/#advancedSearch/default**](https://www.embase.com/#advancedSearch/default)**, select Advanced search, place the**

**entire search string in the box, use all default settings.**

('pediatrics'/exp OR 'pediatrics' OR 'child'/exp OR 'child' OR 'infant'/exp OR 'infant' OR 'adolescent'/exp OR 'adolescent' OR pediatric*:ti,ab OR paediatric*:ti,ab OR child*:ti,ab OR infant:ti,ab OR baby:ti,ab OR teen*:ti,ab OR adolescen*:ti,ab) AND ('telemedicine'/exp OR 'telemedicine' OR 'remote consultation'/exp OR 'remote consultation' OR telemedicine:ti,ab OR 'video consultation':ti,ab OR telehealth:ti,ab OR 'remote consultation':ti,ab) AND ('covid 19'/exp OR 'covid 19' OR 'sars cov 2'/exp OR 'sars cov 2' OR 'severe acute respiratory syndrome coronavirus 2'/exp OR 'severe acute respiratory syndrome coronavirus 2' OR ncov OR '2019 ncov'/exp OR '2019 ncov' OR 'coronavirus' OR 'coronavirus'/exp OR coronavirus OR cov) AND ('patient satisfaction'/exp OR 'patient satisfaction' OR satisfaction:ti,ab OR attitude*:ti,ab OR perception*:ti,ab) AND ([article]/lim OR [article in press]/lim)

**CINAHL (Housed in EBSCO)**

**Steps: In the subscription-only database, select Advanced search, add an additional**

**search box since there are 4 strings, place each of the 4 strings separately in each search**

**box, and use all default options without selecting specific fields.**

((((((MH Pediatrics+)) OR ((MH Child+)) OR ((MH Infant+)) OR ((MH Adolescent+)) OR ((TI pediatric* OR AB pediatric*)) OR ((TI paediatric* OR AB paediatric*)) OR ((TI child* OR AB child*)) OR ((TI infant OR AB infant)) OR ((TI baby OR AB baby)) OR ((TI teen* OR AB teen*)) OR ((TI adolescen* OR AB adolescen*))))

AND

((((MH Telemedicine+)) OR ((MH "Remote Consultation+")) OR ((TI telemedicine OR AB telemedicine)) OR ((TI "video consultation" OR AB "video consultation")) OR ((TI "remote consultation" OR AB "remote consultation")) OR ((TI telehealth OR AB telehealth)) OR ((TI "remote consultation" OR AB "remote consultation")))))

AND

(COVID-19 OR (MH COVID-19+) OR SARS-CoV-2 OR (MH sars-cov-2+) OR "Severe Acute Respiratory Syndrome Coronavirus 2" OR NCOV OR "2019 NCOV" OR ((MH coronavirus+) OR coronavirus OR COV )))

AND

((((((MH "Patient Satisfaction+")) OR ((TI satisfaction OR AB satisfaction))) OR ((TI attitude* OR AB attitude*))) OR ((TI attitude* OR AB attitude*))) OR ((TI perception* OR AB perception*)))

**PsycINFO (Housed in EBSCO)**

**Steps: In the subscription-only database, select Advanced search, add an additional**

**search box since there are 4 strings, place each of the 4 strings separately in each search**

**box, and use all default options without selecting specific fields.**

(DE "Pediatricians" OR DE "Pediatrics") OR (DE "Child Health") ) OR TI ( pediatric* OR paediatric* OR baby OR babies OR toddler* OR infant* OR child* OR teen* OR adolescent* ) OR AB ( pediatric* OR paediatric* baby OR babies OR toddler* OR infant* OR child* OR teen* OR adolescent* )

AND

DE "Coronavirus" OR DE "COVID-19" OR TI ( "COVID-19" OR "SARS-CoV-2" OR "Severe Acute Respiratory Syndrome Coronavirus 2" OR "NCOV" OR "2019 NCOV" OR "coronavirus" OR "COV" ) OR AB ( "COVID-19" OR "SARS-CoV-2" OR "Severe Acute Respiratory Syndrome Coronavirus 2" OR "NCOV" OR "2019 NCOV" OR "coronavirus" OR "COV" )

AND

(DE "Telemedicine" OR TI ( telemedicine OR "remote consultation" OR "video consultation" OR telehealth ) OR AB ( telemedicine OR "remote consultation" OR "video consultation" OR telehealth )

AND

(DE "Satisfaction") OR (DE "Health Attitudes") ) OR TI ( satisfaction OR attitude* OR perception* ) OR AB ( satisfaction OR attitude* OR perception* )

Filter: Academic journals

^The search strings were first developed for the PubMed database and then translated so that they were identical/as similar as possible for the CINAHL and Embase databases using the Polyglot Search in the Systematic Review Accelerator developed by the Institute for Evidence-Based Health Care (https://sr-accelerator.com/). The Polyglot Search in Systematic Review Accelerator did not allow to translate a search string for the PsycInfo database in EBSCO from the PubMed search string; instead, the search string for the CINAHL database was translated so that it was identical/as similar as possible to the PsychInfo database search using the Polyglot Search in the Systematic Review Accelerator.
