# Supplementary material for: Satisfaction With Telehealth Services Compared With Nontelehealth Services Among Pediatric Patients and Their Caregivers: Systematic Review of the Literature
Source: JMIR Pediatr Parent. 2023 Apr 27;6:e41554. doi: 10.2196/41554 (PMC10176140; doi:10.2196/41554)
Supplement: Multimedia Appendix 3 [file pediatrics_v6i1e41554_app3.docx]

**Multimedia Appendix 3.** Quality of evidence in the 14 studies included in the systematic review including study design, quality of evidence rating based on type of design only, differences in socio-demographic characteristics of participants in the telehealth and non-telehealth comparison groups, and reliability and validity of data collection tools

| **Study** | **Study**  **design** | **Quality of evidence based on type of design only*** | **Similarities/differences in socio-demographic characteristics of participants in the non-telehealth and telehealth comparison groups** | **Satisfaction data collection tools:**  **reliability and validity** |
| --- | --- | --- | --- | --- |
| Corona et al., 2021 [14] | **Quasi-experimental study with 3 groups** | Moderate | The participants included in the comparisons were similar in terms of age but different in terms of race/ethnicity (74% White for in-person visits, 54% White for telemedicine visits, 70% for hybrid visits). | The satisfaction surveys were developed by the authors. There was no information on the reliability and validity of the surveys. |
| Hoi et al., 2022 [15] | **Cohort study (prospective)** | Low to Moderate | The two groups (telehealth and in-person) were similar in terms of age and sex (e.g. 57% male in the in-person group and 58% male in the telehealth group). | Reliability and validity of satisfaction surveys were not presented. All patients who completed a care encounter were invited to complete a Press Ganey survey on satisfaction. Results were summarized as caregivers having positive sentiments or negative sentiments towards the telehealth visit or the in-person visit. Specific responses to each item of the survey were not presented. Two reviewers analyzed the data from the surveys independently. |
| Holzman et al., 2021 [16] | **Case-control study (retrospective)** | Low to Moderate | Propensity score matching was used to separate patients into the two groups (in-person and telehealth). The two groups (telehealth and in-person) were similar in terms of age, language and type of visit but not in terms of sex. | All patients who completed a care encounter were invited to complete a reliable and valid survey on satisfaction. The survey was administered by an outside entity (NRC Health). |
| Johnson et al., 2020 [17] | **Analytical cross-sectional study (retrospective)** | Low | There was no information on socio-demographic characteristics for the two groups (telehealth and in-person). | No information on the reliability and validity of the survey was available. |
| Katz et al., 2021 [18] | **Analytical cross-sectional study (retrospective)** | Low | The two groups (telehealth and in-person) differed in patient age and sex. | The authors used a reliable and valid survey: the Hospital Consumer Assessment of Healthcare Providers and Systems Survey. The survey was administered through an outside entity (Press Ganey). |
| Kennelly et al., 2021 [19] | **Analytical cross-sectional study (retrospective)** | Low | Information on socio-demographic characteristics of the two groups (in-person and telehealth) was not presented. | A reliable and valid survey was administered through an outside entity (Press Ganey). |
| Love et al., 2022 [20] | **Cohort study (prospective)** | Low to Moderate | Comparisons of socio-demographic characteristics of participants in the in-person and telehealth groups were not presented. | Reliability and validity of satisfaction surveys were not presented. Information on how the survey was developed was not available. All patients who completed a care encounter were invited to complete an oral survey on satisfaction. Specific responses to each item of the survey 2 weeks after the visit were not presented. |
| Mahmoud et al., 2022 [21] | **Analytical cross-sectional study (retrospective)** | Low | The two groups (telehealth and in-person) were similar in terms of sex but not in terms residence (66.4% of participants in the virtual group were rural residents compared to 47.7% in the in-person group). | The authors used a Patient Experience Assessment Form. The survey was administered through an outside entity (Press Ganey). Information on the reliability and validity of the survey was not presented. |
| Marques et al., 2022 [22] | **Analytical cross-sectional study (retrospective)** | Low | Comparisons of socio-demographic characteristics of participants in the in-person and telehealth groups were not presented. | The authors used a validated 16-item patient experience survey used at multiple healthcare systems throughout the USA. The survey was administered through an outside entity (Press Ganey). Only top box responses on the survey were provided. |
| McCoy et al., 2022 [23] | **Cohort study (prospective)** | Low to Moderate | The two groups (telehealth and in-person) were similar in terms of race/ethnicity (94.7% White in the in-person group and 94.9% White in the telehealth group) but not in sex (61.9% male in the in-person group and 49.2% male in the telehealth group). | Reliability and validity of satisfaction surveys were not presented. All patients who completed a care encounter were invited to complete a survey on satisfaction. Results were summarized mean scores for the different satisfaction survey items. Specific responses to each item of the survey were presented both in a table and graph. |
| Mustafa et al., 2021 [24] | **Cohort study (prospective)** | Low to moderate | The three groups (telehealth, telephone, and in-person) differed in age, sex, and primary diagnosis. | Reliability and validity of satisfaction surveys were not presented. Information on how the survey was developed was not available. Specific responses to surveys items were presented. |
| Ragamin et al., 2021 [25] | **Analytical cross-sectional study (retrospective)** | Low | Comparisons of socio-demographic characteristics of participants in the in-person and telehealth groups were not presented. | The Patient Satisfaction Survey was reliable and valid. |
| Summers et al., 2022 [26] | **Analytical cross-sectional study (retrospective)** | Low | Comparisons of socio-demographic characteristics of participants in the in-person and telehealth groups were not presented. | A Patient Experience survey was conducted by an outside entity (NRCHealth). Reliability and validity of the survey were not discussed. Patient satisfaction scores were presented for “recommend institution” and recommend provider” for in-person and telehealth visits. |
| Troncone et al., 2022 [27] | **Cohort study (prospective)** | Low to moderate | The two groups (telehealth and in-person) had similar characteristics in terms of age and duration of diabetes. | Reliable and valid surveys were used such as the Comprehensive assessment of satisfaction with care (Cronbach’s alpha=0.973) and the Jefferson scale of patient perceptions of physician empathy (Cronbach’s alpha=0.896). |

*Quality of evidence rating is based on the Grading of Recommendations, Assessment, Development, and Evaluation Criteria for initial study rating:

Randomized-controlled trial: high rating

Quasi-experimental pre-post test study: moderate rating

Cohort study (prospective) and case-control study (retrospective): Low to moderate rating

Analytical cross-sectional study (retrospective): low rating
