# Supplementary material for: Satisfaction With Telehealth Services Compared With Nontelehealth Services Among Pediatric Patients and Their Caregivers: Systematic Review of the Literature
Source: JMIR Pediatr Parent. 2023 Apr 27;6:e41554. doi: 10.2196/41554 (PMC10176140; doi:10.2196/41554)
Supplement: Multimedia Appendix 4 [file pediatrics_v6i1e41554_app4.docx]

Multimedia Appendix 4. Quality of evidence scores based on JBI critical assessment tool common questions

|  | Were the criteria for inclusion in the sample clearly defined? | Were the study subjects and setting described in detail? | Were the comparison groups (telehealth and non-telehealth) comparable in terms of socio-demographic characteristics? | Was the time period (such as months and years) for visits clearly defined? | Was satisfaction measured in the same way in the comparison groups? | Were satisfaction measures valid and reliable? | Were appropriate statistical analyses used? | **Total score** |
| --- | --- | --- | --- | --- | --- | --- | --- | --- |
| Corona et al., 2021 [14] | 1 | 1 | 0.5 | 1 | 1 | 0 | 1 | **5.5** |
| Hoi et al., 2022 [15] | 1 | 1 | 1 | 1 | 1 | 1 | 1 | **7** |
| Holzman et al., 2021 [16] | 1 | 1 | 0.5 | 1 | 1 | 1 | 1 | **6.5** |
| Johnson et al., 2020 [17] | 1 | 0.5 | 0 | 1 | 1 | 0 | 1 | **4.5** |
| Katz et al., 2021 [18] | 1 | 0.5 | 0 | 1 | 1 | 1 | 1 | **5.5** |
| Kennelly et al., 2021 [19] | 1 | 0.5 | 0 | 1 | 1 | 0 | 1 | **4.5** |
| Love et al., 2022 [20] | 1 | 0.5 | 0 | 1 | 0.5 | 0 | 1 | **4** |
| Mahmoud et al., 2022 [21] | 1 | 1 | 0.5 | 1 | 1 | 1 | 1 | **6.5** |
| Marques et al., 2020 [22] | 1 | 0.5 | 0 | 1 | 1 | 1 | 1 | **5.5** |
| McCoy et al., 2022 [23] | 1 | 1 | 0.5 | 1 | 1 | 1 | 1 | **6.5** |
| Mustafa et al., 2021 [24] | 1 | 0.5 | 0 | 1 | 1 | 1 | 1 | **5.5** |
| Ragamin et al., 2021 [25] | 1 | 0.5 | 0 | 1 | 1 | 0 | 1 | **4.5** |
| Summers et al., 2022 [26] | 1 | 0.5 | 0 | 1 | 1 | 1 | 1 | **5.5** |
| Troncone et al., 2022 [27] | 1 | 1 | 1 | 1 | 1 | 1 | 1 | **7** |

1=Yes, 0.5=Partially, 0=No/unclear
